# Spontaneous rupture of pyogenic liver abscess with subcapsular hemorrhage mimicking ruptured hepatocellular carcinoma: A case report

**DOI:** 10.1097/MD.0000000000025457

**Published:** 2021-04-16

**Authors:** Nan Seol Kim, Hea Rim Chun, Hae Il Jung, Jin Ku Kang, Sul Ki Park, Sang Ho Bae

**Affiliations:** a Department of Anesthesiology and Pain Medicine; b Department of Surgery, Soonchunhyang Uiversity Cheonan Hospital, Soonchunhyang University College of Medicine, Cheonan-si, Chungcheongnam-do, Republic of Korea.

**Keywords:** liver abscess, spontaneous rupture

## Abstract

**Rationale:**

Spontaneous rupture of PLA (pyogenic liver abscess) is an extremely rare and life-threatening event. Ruptured PLA is very difficult to distinguish from malignant HCC (hepatocellular cancer) rupture or cholangiocarcinoma rupture on CT (computed tomography) scan.

**Patient concerns:**

We describe the case of a 71-year-old man with fever, right upper abdominal pain, nausea with intermittent vomiting, and general fatigue. He had no medical or surgical history.

**Diagnosis:**

CT scan showed a hypodense mass in right hepatic lobe and MRI (magnetic resonance imaging) revealed a heterogenous mass of ∼6 cm in segment VI of the liver and heterogenous fluid in the subcapsular region. We made a tentative diagnosis of HCC rupture with subcapsular hemorrhage based on these findings.

**Intervention:**

After improving the patient's condition by administering empirical therapy consisting of intravenous antibiotics and fluids, we performed surgical exploration. Gross examination of the abdomen showed that almost the entire right hepatic lobe was hemorrhagic and affected by peritonitis. Therefore, we performed right hepatectomy. The intraoperative frozen biopsy revealed suspicious PLA with marked necrosis, neutrophil infiltration, and hemorrhagic rupture, although no malignant tissue or fungus was observed. The postoperative secondary pathology report confirmed the diagnosis of PLA with hemorrhagic rupture.

**Outcomes:**

The patient was discharged 13 days after the operation. Follow-up CT was performed 5 months after discharge and revealed no abnormal findings.

**Lessons:**

A high index of suspicion is key to preventing misdiagnosis of ruptured PLA and improving prognosis. Furthermore, even if rupture of the PLA is initially localized, delayed peritonitis may occur during medical treatment. Therefore, vigilant monitoring is essential.

## 1 Introduction

Pyogenic liver abscess (PLA), a suppurating infection of the hepatic parenchyma, is a serious, life-threatening condition. Recent advances in imaging techniques and treatment modalities have markedly improved outcomes for most patients with PLA. Nevertheless, PLA remains a potentially fatal disease with a mortality rate of 10% to 15%.^[1,2]^ Complications of PLA can include pleural effusion, abscess rupture, peritonitis, septic shock, endogenous endophthalmitis, metastatic central nervous system infection, and psoas abscess.^[3]^

Spontaneous rupture of PLA is an extremely rare and life-threatening event. Early diagnosis and treatment of PLA are critical for patient management, but the clinical presentation may be subtle and nonspecific (abdominal pain, fever, nausea, and vomiting). Liver function tests can be more or less abnormal depending on the extent of the abscess, the cause of the abscess, and the severity of sepsis. The diagnosis of PLA is based mainly on computed tomography (CT) findings.^[1,4]^ Unfortunately, CT is a sensitive but often nonspecific test for detecting hepatic abscess. A biopsy is usually necessary for microbiologic confirmation of the diagnosis.^[4]^ It is particularly difficult to differentiate PLA rupture from malignant hepatocellular carcinoma (HCC) rupture or cholangiocarcinoma rupture using only CT.^[5]^ In patients who have not previously undergone any invasive procedures for the treatment of PLA, the diagnosis of spontaneous rupture of PLA is based on radiologic or gross findings of ruptured PLA during surgery.^[3]^

We herein describe a case of spontaneous rupture of PLA with subcapsular hemorrhage mimicking HCC and causing delayed peritonitis.

## 2 Case report

Written informed consent was obtained from the patients for the publication of this report. A 71-year-old man presented to the emergency room (ER) with fever, right upper abdominal pain, nausea with intermittent vomiting, and general fatigue over the past 4 days. He had no medical or surgical history. He initially visited local clinics and underwent ultrasonography. He was referred to our hospital upon completion of ultrasonography.

Physical examination of the patient revealed fever (38.4°C), tachycardia (114 beats/min), tachypnea (22 breaths/min), and a blood pressure of 100/60 mm Hg. There was no clinical jaundice. The patient reported pain in the upper abdominal regions on palpation. Signs of peritoneal irritation were absent.

Blood count analysis showed a hemoglobin level of 13.7 g/dL and an elevated white blood cell count of 11,660/μL (75% neutrophils) and thrombocytopenia (platelet count: 105,000/μL). The serum level of C-reactive protein was markedly elevated at 343.34 mg/L. Although serum levels of alkaline phosphatase (ALP) and gamma-glutamyltranspeptidase were normal, levels of aspartate aminotransferase (1216 U/L) and alanine aminotransferase (815 U/L) were markedly elevated. Total bilirubin and albumin levels were within normal limits, and the estimated glomerular filtration rate was slightly decreased (41.76 mL/min). Serological tests for hepatitis B and C were negative. In addition, the levels of tumor markers, alpha-fetoprotein, and carcinoembryonic antigen were normal, and amebiasis serology was negative. A coagulation test showed a slightly prolonged clotting time (international normalized ratio: 1.28). We did not isolate any organisms from the blood culture collected in the ER before antibiotic use.

The plain chest and abdominal X-rays were normal and showed no pneumoperitoneum.

**Figure 1:**
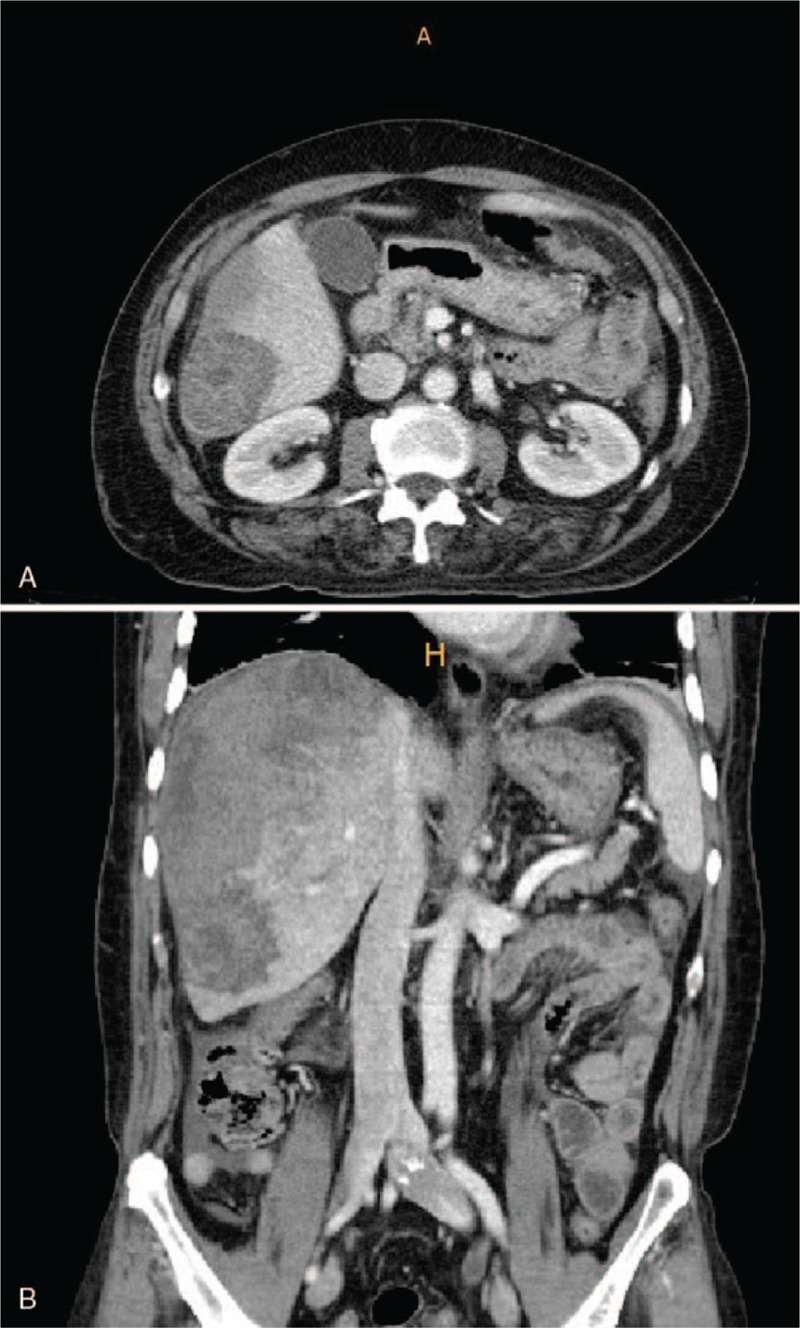
Computed tomography showed a hypodense mass in segment VI of the right hepatic lobe. There is no pneumoperitoneum.

A CT scan of the abdomen showed a hypodense mass in segment VI of the right hepatic lobe. The initial interpretation was a hematoma caused by HCC rupture (Fig. 1). However, the levels of tumor markers, alpha-fetoprotein, and carcinoembryonic antigen were normal. In addition, the patient had no history of hepatitis, cirrhosis, or recent trauma. Therefore, magnetic resonance imaging (MRI) was performed to provide additional information for the differential diagnosis.

**Figure 2:**
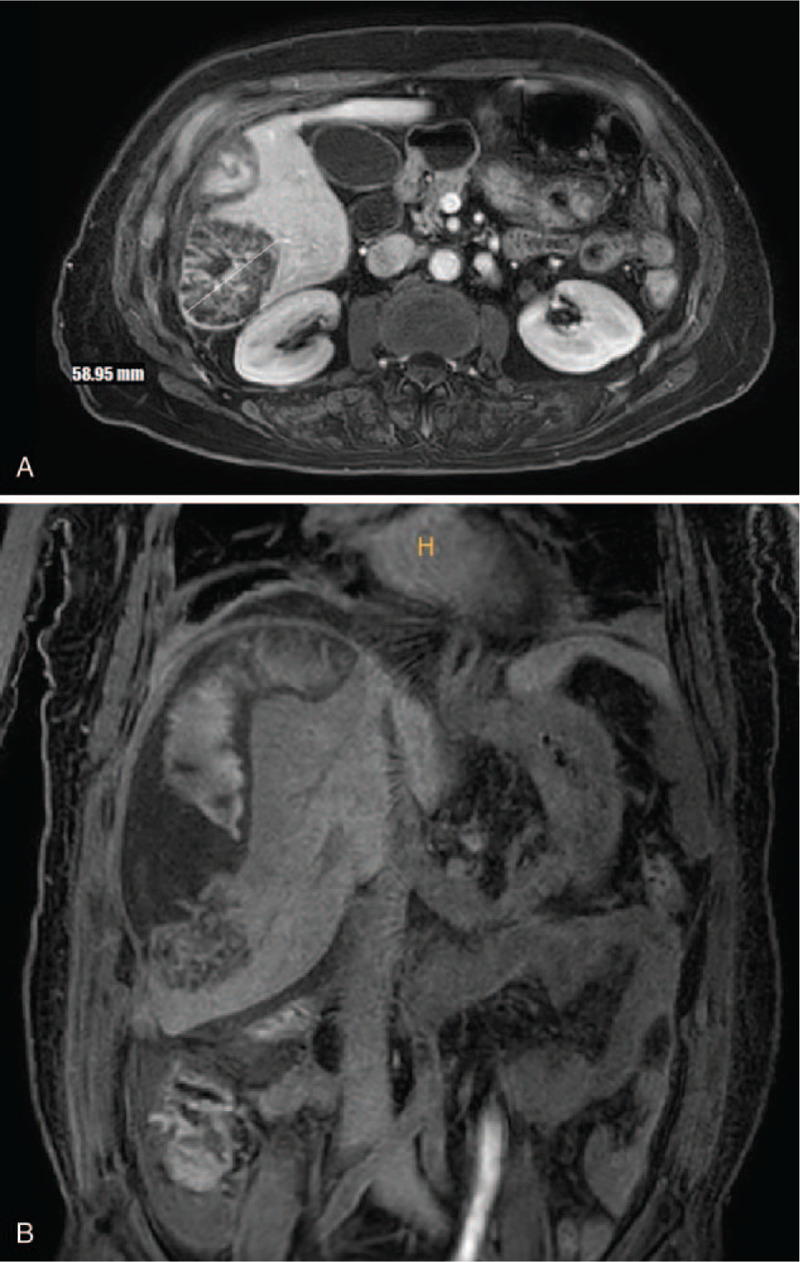
MRI showed heterogenous mass of ∼6 cm in segment VI of the liver and heterogenous fluid in the subcapsular region.

MRI was performed the day after the patient was admitted to the hospital. Imaging showed a heterogenous mass of approximately 6 cm in segment VI of the liver and heterogenous fluid in the subcapsular region (Fig. 2). We made a tentative diagnosis of HCC rupture with subcapsular hemorrhage based on these findings. We suspected that the fever was caused by hematoma reabsorption or secondary infection. We decided to perform surgical resection of the liver after improving the patient's condition by administering empirical therapy consisting of intravenous antibiotics and fluids.

Three days after the patient was admitted to the hospital, his fever subsided, and his white blood cell count was normalized, but severe abdominal pain persisted.

Five days after the patient was admitted, we performed surgical exploration under general anesthesia with tracheal intubation. We made J-shaped subcostal incisions and opened the abdominal cavity layer by layer. Gross examination of the abdomen showed that almost the entire right hepatic lobe was hemorrhagic and affected by peritonitis. Therefore, we performed a right hepatectomy. The operating time was 3 h 40 min, and the intraoperative hemorrhage volume was 1100 mL. The intraoperative frozen section biopsy revealed suspicious PLA with marked necrosis, neutrophil infiltration, and hemorrhagic rupture, although no malignant tissue or fungus was observed. The postoperative secondary pathology report confirmed the diagnosis of PLA with hemorrhagic rupture. Based on these findings, the patient was re-diagnosed with ruptured PLA.

The patient was discharged 13 days after the operation. Follow-up CT was performed 5 months after discharge and revealed no abnormal findings. The results of routine blood tests were normal. In addition, colonoscopy was performed and showed normal findings.

## 3 Discussion

PLA is a rare condition. Its annual incidence ranges from 3.6 to 17.6 per 1,000,000 people, with significant geographic variation.^[6]^ The incidence of PLA appears to increase with age and the presence of comorbidities.^[6,7]^ Common risk factors for PLA include diabetes mellitus, underlying hepatobiliary disease, and gastrointestinal malignancy.^[7]^

PLA is presumed to be secondary to biliary tract disease in patients with cholecystitis, cholangitis, or documented biliary duct abnormalities. However, up to 55% of patients present with “cryptogenic” PLA, meaning that there is no obvious source of infection.^[2,3]^ In patients with chronic cholecystitis, there may be contiguous spread of sepsis from lower segment IV and/or segment V to the liver parenchyma if the infected gallbladder is perforated.^[1]^ Therefore, chronic cholecystitis was excluded in our patient. The cause of our patient's PLA was unclear.

Common laboratory abnormalities include leukocytosis, hypoalbuminemia, prolonged prothrombin time, and elevated inflammatory markers. An elevated ALP level is the most commonly observed laboratory abnormality, occurring in up to 90% of patients.^[7]^ However, laboratory findings are nonspecific for PLA diagnosis. In our patient, the ALP level was within the normal range.

Of PLA cases in which blood or pus culture studies were performed, only 53% produced positive microbiologic reports.^[2]^ In addition, elderly PLA patients appear to have a slightly higher rate of negative reports (i.e., no growth) in both blood and pus cultures (as in the case described in the present study). Thus, diagnosing PLA can be challenging in the geriatric population.^[8]^

As stated previously, a ruptured PLA is very difficult to distinguish from malignant HCC rupture or cholangiocarcinoma rupture on a CT scan.^[5]^ Therefore, differential diagnosis requires microbiologic examination or MRI. However, as our case study illustrates, accurate diagnosis can be difficult even with MRI. In addition, superinfection of primary and metastatic liver malignancies is possible. Therefore, it is important not to miss underlying malignant disease when faced with PLA.^[1]^

Spontaneous rupture of HCC is a potentially life-threatening complication. Its diagnosis may be difficult in patients without a history of cirrhosis or HCC. In 75% of cases, HCC rupture is confirmed by CT or ultrasonography or both. The best treatment approach for ruptured HCC is debatable. The primary goals of treatment are to correct hypovolemic shock and stabilize the patient.^[9]^ Hemodynamically stable patients with no active bleeding should be managed conservatively and then given definitive treatment (e.g., liver resection or transarterial chemoembolization). Early partial hepatectomy within 8 days of ruptured HCC is associated with a significantly better survival compared with non-surgical treatment.^[10]^ Therefore, in the present case, we had initially decided to perform surgical resection within 8 days of hospital admission.

Percutaneous drainage and long-term antibiotic therapy are now cornerstones of PLA treatment and have high levels of safety and efficacy.^[11]^ However, surgical treatment is still necessary in some cases. The main indications for surgical management are rupture of PLA with peritonitis, inappropriate local or failed percutaneous drainage, multiloculated or septate abscesses, multiple abscesses, and PLA with solid content.^[6]^

Jun et al showed that cirrhosis, large abscesses (>6 cm), gas-forming abscesses, and other septic metastases in patients with PLA are risk factors for spontaneous rupture of PLA.^[3]^ In our patient's case, the PLA was approximately 6 cm in diameter. Generally, ruptures resulting in peritonitis require urgent **s**urgical interventions. Localized ruptures can be managed with drainage, either percutaneous or surgical, in addition to appropriate antimicrobial treatment.^[3]^ In our patient's case, delayed peritonitis occurred while preparing for right hepatectomy with intravenous fluid and antibiotics. This demonstrates that peritonitis may occur during medical treatment even when the patient does not initially present with peritonitis. Therefore, patients should be monitored closely.

It has been suggested that cryptogenic PLA is a sign of occult gastrointestinal malignancy.^[12]^ Therefore, our patient underwent colonoscopy 5 months after discharge. We did not find incidental colon cancer or high-grade dysplasia.

**In conclusion**, spontaneous rupture of PLA is difficult to diagnose based on clinical symptoms and imaging, including MRI. In addition, incorrect diagnosis may lead to potentially fatal complications. A high index of suspicion is key to preventing misdiagnosis of ruptured PLA and improving prognosis. Furthermore, even if rupture of the PLA is initially localized, delayed peritonitis may occur during medical treatment. Therefore, vigilant monitoring is essential.

## Author contributions

**Conceptualization:** Nan Seol Kim, Sang Ho Bae.

**Writing – original draft:** Nan Seol Kim.

**Writing – review & editing:** Nan Seol Kim, Hea Rim Chun, Hae Il I Jung, Jin Ku Kang, Sul Ki Park, Sang Ho Bae.
